# Effects of continuous renal replacement therapy on Apache-II score, creatinine, and urea nitrogen Levels in patients with acute kidney injury

**DOI:** 10.12669/pjms.39.1.6591

**Published:** 2023

**Authors:** Huihui Hou, Lingzhi Li

**Affiliations:** 1 Huihui Hou, Department of Intensive Care Unit, Shandong Provincial Third Hospital, Cheeloo College of Medicine, Shandong University No.11, Wuyingshan Middle Road, Jinan 250031, Shandong Province, P.R. China; 2 Lingzhi Li, Department of Intensive Care Unit, Shandong Provincial Third Hospital, Cheeloo College of Medicine, Shandong University No.11, Wuyingshan Middle Road, Jinan 250031, Shandong Province, P.R. China

**Keywords:** Continuous renal replacement therapy, Serum electrolytes, Apache score II, Acute kidney injury

## Abstract

**Objective:**

To analyze the effects of continuous renal replacement therapy (CRRT) on acute physiology and chronic health scoring system II (APACHE-II) score, creatinine, and urea nitrogen levels in patients with acute kidney injury (AKI).

**Methods:**

Medical records of 79 patients with AKI treated in Shandong Provincial Third Hospital from January 2019 to January 2021 were retrospectively divided into two groups based on the received treatment. Of them 37 patients received intermittent hemodialysis (IHD) treatment (control group) and 42 patients received CRRT (observation group). Clinical efficacy, survival rate, severity of disease, renal function and serum electrolytels and fluid balance were analyzed.

**Results:**

After the treatment, the total efficacy of the observation group was 95.24%, and the 6-month survival rate was 97.62%, which was higher than 81.08% and 83.78% in the control group, respectively (*P*<0.05). The Apache-II score of the observation group was (15.76±4.29), which was lower than that of the control group (23.62±5.37). Levels of creatinine, urea nitrogen, and serum levels of potassium (K^+^), chlorine (Cl-) and sodium (Na^+^) in the observation group were lower than those in the control group (*P*<0.05).

**Conclusion:**

CRRT can achieve significant results in the treatment of patients with AKI, help to improve the curative effect, survival rate, alleviate the severity of the disease, recovery of renal functions, the recovery of serum electrolytels and fluid balance.

## INTRODUCTION

Acute kidney injury is characterized by a rapid deterioration of kidney function that affects the balance of pH and electrolytes and leads to the retention of metabolic waste products. It is a common clinical problem in the intensive care unit, and is associated with high mortality.^1^,^2^ AKI is often accompanied by symptoms such as hyper-catabolism, abnormal hemodynamics, brain and pulmonary edema, which aggravates the degree of inflammatory reaction and significantly reduces immune function. Timely treatment, therefore, is essential in preventing mortality of patients.^3^ Hemodialysis is an effective measure for treatment of patients with AKI. It ensures timely excretion of metabolites, such as urea nitrogen and creatinine, and allows to effectively maintain fluid balance and prevent electrolyte and acid-base disturbances.^4^

However, studies have found that hemodialysis treatment is associated with cardiovascular instability and significant fluctuations in the levels of metabolites and electrolytes^5^ With the recent developments in medical technology, CRRT has been gradually introduced as a treatment option for patients with AKI. CRRT is a continuous and long-term in vitro blood purification for 24 hours or nearly 24hours/day that has the advantages of being a highly safe and effective method.^6^ It allows timely and efficient removal of inflammatory mediators, thus restoring electrolyte balance.^7^

In the current study, we use clinical efficacy, survival rate, disease severity, renal function level and serum electrolytels and fluid balance as evaluation indexes to retrospectively analyze the application effect of CRRT in treating patients with AKI.

## METHODS

A total of 79 patients medical records with AKI treated in Shandong Provincial Third Hospital from January 2019 to January 2021 were retrospectively selected. The study protocol was approved by the hospital ethics committee (Approval number: 20211224, Date: 2021-12-17). All patients received symptomatic treatment such as nutritional support, blood volume supplementation, anti-infection care, and serum electrolyte correction. Patients were retrospectively divided into two groups based on the treatment. The control group included 37 patients that received intermittent hemodialysis (IHD). The primary diseases in the control group were hypertensive nephropathy (n=11), diabetic nephropathy (n=11), chronic interstitial nephritis (n=7) and chronic glomerulonephritis (n=8). Patients (n=42) that received CRRT comprised the observation group. The primary diseases in the observation group were hypertensive nephropathy (n=12), diabetic nephropathy (n=16), chronic interstitial nephritis (n=8) and chronic glomerulonephritis (n=6). The two groups are comparable as there were no significant differences in baseline data such as gender (*χ^2^*=0.768, *P*>0.05), age (*t*=0.750, *P*>0.05), and primary disease (*t*=1.009, *P*>0.05) between the two groups.

***IHD was performed as follows:*** dialysis was done using Box10101dialysis machine (Gambro Lundia AB) with the hemodialysis filter Prismaflex M100 (Gambro industries) and bicarbonate solution. The parameters were as follows: transmission area 1.3m^2^, ultrafiltration coefficient 40ml/minute, dialysate flow 500ml/minute, 4~5 hours each time, 2~3 times a week.

***The CRRT was performed as follows:*** hemodialysis was done using FH55 filter, PRISMA system hemodialysis machine and FH66d blood filter, replacement fluids of improved Port. The venous site on the right side of the neck was located, the double lumen catheter was indwelled by Seldinger technology, the blood flow was 250mL/minute; the synchronous input mode was set, and sodium bicarbonate replacement solution was administered by the pre-dilution method. The daytime treatment was set for 10~12 hours, the infusion volume of sodium bicarbonate replacement fluids was 16~24L, and input was 2~4 circulating replacement fluids. After starting the treatment, anticoagulation was performed by the minimum heparinization method. The initial dosage was 0.3~0.5 IU/kg, and the additional dosage was 2~10 IU/hour. It was continuously administered with the infusion pump to prolong the activated prothrombin time by 1.5~2 times. Catheterization was closely monitored, and in case of coagulation the catheter and filter were flushed in a timely manner. According to the specific condition of the patient, the acid-base concentration and electrolyte concentration were adjusted. The blood flow rate was set at 180~220 ml/minute, and the replacement fluids was set to 1~2 L/hour. The ultrafiltration volume was adjusted every 30 minutes or according to the physiological needs, water and sodium retention and treatment volume.

### Inclusion criteria:

- Histopathological examination confirmed AKI;
- Age ranges from 18 to 80 years;
- Good compliance and active cooperation;
- CRRT and IHD was performed for the first time.

### Exclusion criteria:

- History of previous renal transplantation;
- Allergies;
- Coagulation dysfunction;
- Severe hypotension;
- Malignant tumor or mental disorder;
- Cognitive impairment.
- Preexisting chronic kidney disease requiring CRRT or IHD

### Indicators and evaluation of treatment effect:

Medical records of all patients contained basic information and the following indicators after the treatment: 1) treatment effect, as represented by the three levels: remarkable effect (after the treatment, the patient’s condition was significantly improved and most of the indexes of renal function have returned to normal); effective (patient’s condition was gradually improved, and the indexes of renal function also gradually tend to normal); ineffective (no change or even deterioration of the condition).^8^ 2) Survival at six months after the treatment. 3) The severity of the disease before and after the treatment as evaluated by the score of acute physiology and chronic health status scoring system II (APACHE-II), including three items: acute physiology, age and chronic health status before the disease. The total score is 71 points, and the score is directly proportional to the severity of the disease.^9^ 4) Renal function level before and after the treatment. Test method was as follows: 5ml fasting venous blood sample was taken and centrifuged at 3000r/minute×10 minutes. Expression levels of serum creatinine and urea nitrogen were measured in the supernatant by automatic biochemical analyzer (AU680, Beckman Kurt Company). In addition, the level of serum electrolytels and fluid balance, including serum potassium (K^+^), chlorine (Cl-) and sodium (Na^+^), was measured by colorimetry. The reagents were purchased from the Research Institute of Shanghai Naval Medical College.

### Statistical analysis:

SPSS 22.0 was used for data analysis. Descriptive statistics (mean, standard deviation [SD], counts) was used to describe categorical and quantitative variables. Chi-square tests were conducted to compare discrete variables and student’s t-tests was used to compare continuous variables. The clinical efficacy and six-month survival rate were compared using Chi-square tests; the severity of the disease and the level of renal function, and the recovery of serum electrolytes and fluid balance were compared using student’s t-tests. All statistical tests were 2-sided, and statistical significance was set at *P* =0.05.

## RESULTS

Seventy-nine patients were included in this retrospective study. There was no significant difference in general data between the two groups (*P*>0.05), as shown in Table-I. After the treatment, 22 patients in the observation group exhibited remarkable treatment effect, treatment of 18 patients was effective and two cases were ineffective. The total efficacy was 95.24% (40/42), and the 6-month survival rate was 97.62% (41/42). In the control group, treatment of 15 patients was considered remarkably effective, of 15 patients - effective and 17 patients showed no effect of the treatment. The total effective rate was 81.08% (30/37), and the survival rate was 83.78% (31/37). The total efficacy and six-month survival rate of the observation group were higher than those of the control group (*χ^2^*=3.906 and 4.663, *P*=0.048 and 0.031), indicating that the observation group treated with CRRT was better than the control group treated with IHD in terms of clinical efficacy and survival (Table-II).

**Table-I:** Comparison of general information between the two groups[*n*(%),*χ̅*±*S*].

Characteristic	Control group (n=37)	Observational group (n=42)	χ^2^>t	P
***Gender, No. (%)***				
Male	23 (62.16)	22 (52.38)	0.768	0.381
Female	14 (37.84)	20 (47.62)		
Age, mean (SD), y	56.13 (11.73)	57.90 (8.80)	0.750	0.458
***Primary disease, No***				
Hypertensive nephropathy	11	12	1.009	0.799
Diabetic nephropathy	11	16		
Chronic interstitial nephritis	7	8		
Chronic glomerulonephritis	8	6		

**Table-II:** Comparison of the clinical efficacy of the two groups [*n*(%)].

Variable	Control group (n=37)	Observational group (n=42)	χ^2^	P
***Clinical efficacy, No. (%)***				
Significantly effective	15 (40.54)	22 (52.38)		
Efficient	15 (40.54)	18 (42.86)		
Invalid	7 (18.92)	2 (4.76)		
Total effective rate	30 (81.08)	40 (95.24)	3.906	0.048
Six month survival rate, No. (%)	31 (83.78)	41 (97.62)	4.663	0.031

There was no significant difference in the severity and renal function level between the two groups before the treatment (*P*>0.05). Table-III After the treatment, the severity of the disease in both groups was alleviated and the improvement in renal function. Through inter-group comparison, the Apache-II score of the observation group was significantly lower than that of the control group, and the expression of creatinine and urea nitrogen were lower than that of the control group (*P*<0.05) The results showed that the observation group treated with CRRT fared better than the control group treated with IHD in terms of improving the severity of disease course and recovery of renal functions.

**Table-III:** Comparison of the severity of the disease and the level of renal function between the two groups (*χ̅*±*S*).

Variable	Control group (n=37)	Observational group (n=42)	t	P
APACHEII score (score)				
Before therapy	30.40±4.02	31.47±3.48	1.268	0.208
After treatment	22.46±3.27^a^	16.59±2.81^a^	8.569	<0.001
Serum creatinine (μmol/L)				
Before therapy	762.00±109.98	776.00±127.77	0.518	0.606
After treatment	482.78±95.84^a^	329.21±90.46^a^	7.322	<0.001
Blood urea nitrogen (mmol/L)				
Before therapy	40.21±6.43	41.04±6.66	0.562	0.575
After treatment	29.70±5.38^a^	18.52±5.86^a^	8.782	<0.001

***Note:***

a compared with the same group before treatment P<0.05.

There was no significant difference in the levels of serum potassium K^+^, Cl- and sodium Na^+^ between the two groups before the treatment (*P*>0.05) (Table-IV). After the treatment, the serum electrolytels and fluid balance of the two groups recovered significantly, and the levels of serum potassium K^+^, Cl- and sodium Na^+^ in the observation group were lower than those in the control group (*P*<0.05).

**Table-IV:** Comparison of the recovery of serum electrolytels and fluid balance between the two groups (*χ̅*±*S*, mmol/L).

Variable	Control group (n=37)	Observational group (n=42)	t	P
K^+^				
Before therapy	4.71±0.75	4.90±0.0.97	0.972	0.334
After treatment	4.41±0.62^a^	4.07±0.65^a^	2.326	0.023
Cl^-^				
Before therapy	92.56±9.98	91.73±8.74	0.394	0.695
After treatment	87.89±9.41^a^	76.88±7.97^a^	5.627	<0.001
Na^+^				
Before therapy	148.59±11.25	149.21±12.71	0.228	0.820
After treatment	136.19±9.83^a^	121.21±10.73^a^	6.433	<0.001

***Note:***

a compared with the same group before treatment P<0.05.

## DISCUSSION

Our study showed that in patients with AKI CRRT resulted in higher total effective rate and survival rate, lower Apache-II score and lower levels of creatinine, urea nitrogen, serum potassium (K^+^), chlorine (Cl-) and sodium (Na^+^) as compared to patients that received intermittent renal replacement therapy.

The pathogenesis of AKI is complex, and the prognosis is difficult. Deterioration of renal function progresses rapidly over a short period of time, the expression of serum creatinine and urea nitrogen increases fast, and the glomerular filtration rate rapidly decreases.^10^ Research shows that AKI is associated with many complications, such as infection, gastrointestinal bleeding, heart failure and uremia and is mainly manifested in polyuria, oliguria, fatigue, and anuria. Additionally, acute renal failure is accompanied by a significant increase in the levels of medium- and macromolecular substances such as hepatocyte growth factor, tumor necrosis factor, insulin-like growth factor and epithelial cell growth factor. Therefore, the main focus of the treatment is to restore the affected balance and to improve patient’s condition.^11^,^12^

In recent years, with the further development of medical technology and the continuous improvement of blood purification therapy, IHD and CRRT became popular for treatment of patients with AKI. AKI in many patients is accompanied by excessive catabolism, crush syndrome, systemic inflammatory response syndrome, multiple organ failure and sepsis. Due to rapid changes in blood gas parameters, osmolality and volume, dialysate composition and blood membrane reaction, IHD scheme may have hemodynamic stability deviations. As a result, some patients cannot tolerate this type of hemodialysis.^13^ CRRT is developed based on traditional hemodialysis technology, which can effectively maintain the stability of hemodynamics and enhance nutrient supplement.^14^ In this study, the CRRT was used in the treatment of patients with AKI. Our results showed that the total effective rate and survival rate after six months in the observation group were higher than those in the control group (*P*<0.05), indicating that the CRRT is helpful to improve the efficacy and survival rate of patients with AKI. Our results are in agreement with the study of Meersch M et al.^15^ Micarelli D^16^ et al. found that the renal function of patients with severe AKI was seriously affected, and the expression of créatinine and urea nitrogen increased significantly. In our study, Apache-II score, serum creatinine and urea nitrogen expression in the observation group were lower than those in the control group (*P*<0.05), indicating that CRRT treatment is helpful to alleviate the severity and renal function level of patients with AKI, which is consistent with the study of An N et al.^17^ In addition, the levels of serum K^+^, Cl- and Na^+^ in the observation group after the treatment were also lower than those in the control group (*P*<0.05), suggesting that the CRRT can also promote the recovery of serum electrolytes and fluid balance in patients with AKI, in agreement with the study of Tangy et al.^18^

CRRT simulates the process of removing solute and water from the kidney by simulating glomerular filtration. It removes solutes with molecular weight less than 50000 in the form of convection. Therefore, endotoxin and inflammatory mediators can be continuously and fully removed. This, in turn, lowers the risk of renal ischemia and reperfusion, promotes the recovery of renal function, protects the functions of other organs, and alleviates the severity of the disease. Moreover, the CRRT can also regulate the acid-base balance, water electrolyte balance, maintain the stable state of hemodynamics, promote timely removal of small molecular substances such as urea nitrogen, K^+^ and creatinine, improve biochemical indexes, retain macromolecules in body fluid and enhance colloidal osmotic pressure by facilitating adsorption, convection, and dispersion. Alleviating the degree of interstitial edema is helpful to improve microcirculation function and restore positive nitrogen balance, to promote the recovery of serum electrolytels and fluid balance.

### Limitations of the study:

It is a single center study with small sample size, few observation indexes and short follow-up time. Prospective studies with large sample size, increased evaluation indexes and longer follow-up time are needed.

## CONCLUSION

CRRT can achieve significant results in the treatment of patients with AKI, help to improve the curative effect and survival rate, alleviate the severity of the disease, recovery of renal function, the recovery of serum electrolytes and fluid balance, and provide reference for clinicians.

### Authors’ contributions:

**HH** conceived and designed the study.

**HH and LL** collected the data and performed the analysis.

**HH** was involved in the writing of the manuscript and is responsible for the integrity of the study.

All authors have read and approved the final manuscript.
